# Supplementary material for: A Critical View of the Application of the APEX Software (Aqueous Photochemistry of Environmentally-Occurring Xenobiotics) to Predict Photoreaction Kinetics in Surface Freshwaters
Source: Molecules. 2019 Dec 18;25(1):9. doi: 10.3390/molecules25010009 (PMC7017383; doi:10.3390/molecules25010009)
Supplement: Supplementary file 1 [file molecules-25-00009-s001.zip › 3.2.4_gcc-4.4.0.zip!/3.2.4_gcc-4.4.0/3.2.4_gcc-4.4.0/doc/octave/html/interpreter/Function-Support.html]

Function Support - Untitled


Next: Example Codes,
Previous: Matrix Algebra,
Up: Diagonal and Permutation Matrices


---

### 20.3 Functions That Are Aware of These Matrices

This section lists the built-in functions that are aware of diagonal and
permutation matrices on input, or can return them as output. Passed to other
functions, these matrices will in general trigger an implicit conversion.
(Of course, user-defined dynamically linked functions may also work with
diagonal or permutation matrices).

- Diagonal Matrix Functions- Permutation Matrix Functions
